# Yellow pea-based pasta's impacts on the salt intake, glycemic parameters and oxidative stress in healthy individuals: a randomized clinical trial

**DOI:** 10.1038/s41598-024-72290-6

**Published:** 2024-10-07

**Authors:** Mamoru Ito, Joto Yoshimoto, Sho Ishii, Tetsuya Maeda, Yu Wada, Yoshikazu Yonei, Mikiya Kishi, Takahiro Ono

**Affiliations:** 1 Central Research Institute, Mizkan Holdings Co., Ltd., Handa-shi, Aichi Japan; 2 New Business Development, Mizkan Holdings Co., Ltd., Chuo-ku, Tokyo, Japan; 3 Anti-Aging Medical Research Center, Faculty of Life and Medical Sciences, Doshisha University, Kyotanabe-shi, Kyoto Japan; 4 Ueno-Asagao Clinic, Taito-ku, Tokyo Japan

**Keywords:** Health care, Medical research

## Abstract

Pea (*Pisum sativum L.*), a widely cultivated legumes globally, is attracting interest as a functional food owing to its antioxidant properties derived from nutritional components such as polyphenols. We previously reported that yellow pea-based pasta (YPP) aids in controlling blood glucose and enhances the sensitivity to saltiness. This study examined the antioxidant effect of YPP and its effects on the salt intake and postprandial blood glucose levels by simulating a real-life scenario. In this open, parallel-group, randomized controlled trial, 40 healthy adult men and women aged 20–65 years, whose salt intake exceeded the target salt equivalent level of the Japanese dietary intake standard, were allocated to the following groups (n = 20): the group consuming one serving of YPP per day and the group maintaining their regular daily dietary habits. The participants who were allocated to the YPP group showed significantly improved oxidative stress markers (BAP/d-ROMs ratio change: control =  − 0.11, YPP = 0.27, *p* = 0.044; lipid peroxide change: control = 0.11, YPP =  − 0.25, *p* < 0.001) than control participants. The effects on salt intake and blood glucose levels were limited. In conclusion, YPP may serve as a functional staple food that improves oxidative stress.

## Introduction

Pea (*Pisum sativum L.*), cultivated in almost all countries worldwide, is one of the most commonly produced legumes, with a production of 12 million tons in 2021^1^. Peas are attracting attention as a functional food since their nutritional components, such as proteins, minerals, and dietary fiber, are beneficial to human health^2^. Furthermore, due to the presence of polyphenols in pea hulls, especially flavonoids and phenolic acids, peas and pea-based products have a variety of health effects, such as antioxidant and anti-inflammatory effects^3^.

Sodium chloride is essential for humans; however, its excessive intake increases the risk of hypertension, cardiovascular disease, and other disorders^4,5^. Thus, the World Health Organization has set a global target salt intake of < 5 g per day^6^, and the Ministry of Health, Labor and Welfare of Japan has set a target salt intake of < 7.5 g and < 6.5 g for men and women aged ≥ 18 years, respectively^7^. However, the actual mean intake of salt is 10.5 and 9 g for men and women, respectively, indicating that achieving the set targets will be challenging^8^. Moreover, salt has adverse impacts on organs, such as the kidneys, regardless of the blood pressure status^9^. Regarding the underlying mechanism, salt intake enhances the activity of the renin-angiotensin system (RAS) in tissues, which increases active oxygen by increasing the activity of NADPH oxidase^10,11^. Salt intake induces inflammation and oxidative stress in tissues; therefore, reducing salt intake is crucial for promoting antioxidation.

Carbohydrates are also an important energy source for humans to maintain their vital functions. However, excessive increases in the blood glucose levels and chronic hyperglycemia induce oxidative stress^12^. Recently, a strong correlation has been found between postprandial hyperglycemia and cardiovascular events^13^ and the underlying mechanisms that have attracted attention among researchers include the production of advanced glycation end-products (AGEs) due to postprandial hyperglycemia, enhanced polyol metabolism, xanthine oxidase activation, enhanced superoxide production by mitochondria, and enhanced oxidative stress via protein kinase C (PKC) activation^14^. Moreover, AGEs activate intracellular signals through the receptor for AGEs (RAGE) and a crosstalk occurs between the AGE/RAGE system and RAS^15^.

For enabling continuous intake of health-beneficial peas, we have developed a highly palatable pasta-like staple food made exclusively from yellow peas (YPP) and investigated its functionality. We previously reported that YPP enhances the sensitivity to saltiness (salt-sensitivity of the taste buds)^16^, is a low glycemic index (GI) food that does not easily elevate the postprandial blood glucose levels when consumed in a single dose^17^, and that it suppresses postprandial insulin secretion^16^. Based on these characteristics, we hypothesized that continuous intake of YPP may contribute to the reduction of the salt intake level, suppression of chronic hyperglycemia, and oxidative stress reduction. This hypothesis was evaluated in this exploratory study.

## Results

### Participants

**Fig. 1:**
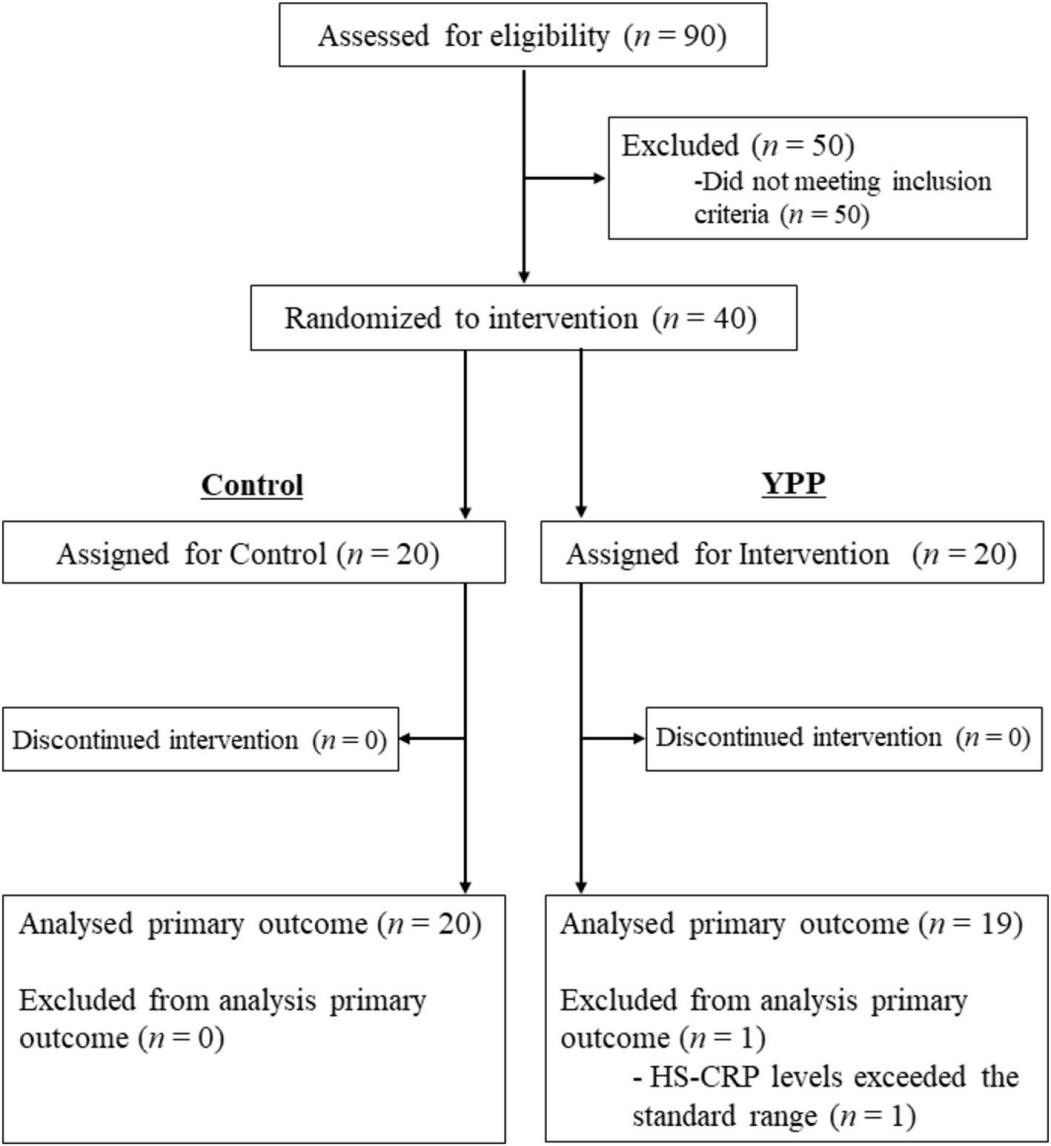
Transition chart of the intervention participants.

Table 1 shows the details of the participants enrolled in this study, and Fig. 1 shows the flow chart of the number of participants in each group. One participant in the YPP group was excluded from the analysis since their high-sensitivity C-reactive protein (HS-CRP) level exceeded the normal range in the test done in Week 0, suggesting the presence of some type of inflammatory reaction. Supplementary Table S1 shows vital sign and body composition measurements for participants not evaluated in this study. Safety was assessed by physician interviews and adverse event determinations. No adverse events attributable to the intervention were identified in this study.

**Table 1:** Baseline characteristics of participants.

	Control (n = 20)		YPP (n = 19)	
Age (years)	47.9	(10.8)	44.8	(10.6)
Sex (n)				
Male	11		10	
Female	9		9	
Weight (kg)	63.9	(12.2)	62.1	(12.3)
BMI (kg/ m^2^)	23.5	(3.10)	22.7	(3.65)
SBP (mmHg)	122	(12.2)	119	(10.9)
DBP (mmHg)	74.7	(11.9)	68.1	(12.6)
Glucose (mg/dL)	93.4	(7.18)	94.6	(7.26)
HbA1c (%)	5.35	(0.38)	5.43	(0.20)
BDHQ dietary salt intake (g/day)	12.8	(4.10)	12.8	(4.08)
Converted 24 h urinary sodium (mEq/day)	152	(23.4)	151.1	(19.6)
BAP (µmol/L)	2391	(187)	2434	(170)
d-ROMs (U.CARR)	384	(93.4)	386	(131)
BAP/d-ROMs	6.51	(1.40)	6.76	(1.61)

Data are presented as mean (SD). BDHQ, Brief-type self-administered diet history questionnaire; BMI, Body mass index; SBP, Systolic blood pressure; DBP, Diastolic blood pressure; HbA1c, Hemoglobin A1c; BAP, Biological antioxidant potential; d-ROMs, Diacron-reactive oxygen metabolites.

### Salt intake level and related biochemical parameters

Table 2 shows the results of the survey on salt intake and sensitivity to saltiness, blood aldosterone levels, and the activity of renin, angiotensin converting enzyme (ACE)1, and ACE2, in Weeks 0 and 4 in the control and YPP groups. Supplementary Table S2 shows the average and total estimated salt intake using self-monitoring devices during the study period. The salt intake level in Week 4 was significantly decreased in both groups (Control, *p* < 0.001; YPP, *p* = 0.035); however, no significant difference in the variation of this parameter was found between the two groups (*p* = 0.27), according to the brief-type self-administered diet history questionnaire (BDHQ) results. The 24-h urine Na^+^ excretion level in Week 4, which was assessed via spot urine collection, was significantly increased in the control group (*p* = 0.036), whereas it did not change significantly in the YPP group (*p* = 0.63). Moreover, no significant difference in the variation of this parameter was found between the two groups (*p* = 0.13, Difference in mean = 17.4, Common SD = 35.7, Effect size: *g* = 0.49, Power: 1 − β = 0.32).

**Table 2:** Salt intake and related biochemical parameters.

	Week 0		Week 4		*p*^1^	Change		*p*^2^
BDHQ dietary salt intake (g/day)								
Control	11.4	(2.21)	9.50	(2.62)	< 0.001***	− 1.94	(2.17)	0.27
YPP	11.8	(3.69)	10.6	(3.93)	0.035*	− 1.15	(2.20)	
Daily urine salt (g/day)								
Control	9.18	(3.16)	9.63	(2.71)	0.59	0.45	(3.66)	0.30
YPP	9.52	(3.48)	8.75	(2.49)	0.36	− 0.77	(3.53)	
Urine salt measured at the clinic (g/day)								
Control	8.05	(1.77)	8.78	(1.72)	0.036*	0.74	(1.46)	0.13
YPP	7.67	(1.62)	7.39	(2.04)	0.63	− 0.29	(2.51)	
Sensitivity to saltiness								
Control	55.1	(15.6)	49.7	(18.9)	0.26	− 5.40	(20.6)	0.96
YPP	56.7	(16.9)	50.9	(16.4)	0.29	− 5.74	(23.0)	
Aldosterone (pg/mL)								
Control	62.3	(37.5)	62.3	(54.4)	0.56	− 0.02	(38.5)	0.63
YPP	104	(154)	108	(132)	0.61	3.86	(95.8)	
Plasma renin activity (ng/mL/h)								
Control	1.12	(0.98)	1.53	(1.08)	0.14	0.41	(0.76)	0.12
YPP	2.16	(2.31)	2.12	(1.76)	0.61	− 0.042	(0.98)	
ACE1 (IU/L)								
Control	13.6	(3.62)	14.9	(4.30)	< 0.001***	1.35	(1.41)	0.65
YPP	14.1	(3.85)	15.6	(4.03)	< 0.001***	1.54	(1.16)	
ACE2 (pg/mL)								
Control	2519	(1423)	2220	(1451)	0.20	− 299	(1010)	0.28
YPP	2152	(1225)	2014	(888)	0.36	− 138	(638)	

Data are presented as mean (SD). Daily urinary salt content was calculated using a self-monitoring device (KME-03). Urinary salt content measured at the clinic was calculated via spot urine collection. ^1^Within-group comparisons (Week 0 vs. Week 4) using paired-samples *t*-test or Wilcoxon signed-rank test. ^2^Group comparison of change using independent-samples *t*-test or Wilcoxon rank-sum test. *p < 0.05, **p < 0.01, ***p < 0.001. ACE, Angiotensin converting enzyme; YPP, Yellow pea pasta.

The ACE1 activity was significantly increased in both groups (Control, *p* < 0.001; YPP, *p* < 0.001), and no significant difference in variation was found between the two groups (*p* = 0.65). Both groups showed no significant changes in the salt intake level, which was estimated using a self-monitoring device, in sensitivity to saltiness, in aldosterone levels, in renin activity, or in ACE2 activity, and no significant differences in the variation of these parameters were found between the two groups.

### Oxidative stress markers

Table 3 shows the levels of oxidative stress-related biomarkers in Weeks 0 and 4 in the control and YPP groups. At Week 4, neither group showed significant changes in the biological antioxidant potential (BAP)/diacron-reactive oxygen metabolites (d-ROMs) ratio; however, the variation of this parameter was significantly greater in the YPP group (*p* = 0.044) than in the control group. Comparing the two time points, the oxidized low-density lipoprotein (Ox-LDL) level was significantly decreased in both groups (Control, *p* < 0.001; Intervention, *p* = 0.016); however, no significant difference in variation was found between the two groups (*p* = 0.085). No significant change in the lipid peroxide (LPO) level was observed in the control group. However, in Week 4, the LPO level was significantly decreased in the YPP group (*p* < 0.001), and the variation of the parameter was significantly higher in the YPP group than in the control group (*p* < 0.001). Moreover, neither group showed significant changes in the levels of BAP, d-ROMs, 8-hydroxy-2′-deoxyguanosine (8-OHdG), 15-Isoprostane F2t (Isoprostane), or hexanoyl lysine (HEL), and no significant difference in the variation of these parameters was found between the two groups. Of these parameters, the variation in d-ROMs and 8-OHdG was compared between the two groups by t-test, and the effect size and power were as follows; d-ROMs (Difference in mean = 20.1, Common SD = 36.2, Effect size: *g* = 0.56, Power: 1 − β = 0.40), 8-OHdG (Difference in mean = 1.33, Common SD = 2.41, Effect size: *g* = 0.55, Power: 1 − β = 0.40). The results of the BAP/d-ROMs ratio and LPO level measurement indicated that the YPP intake for four weeks improved the oxidative stress level in the participants.

**Table 3:** Oxidative stress markers.

	Week 0		Week 4		*p*^1^	Change		*p*^2^
BAP (μmol/L)								
Control	2558	(143)	2623	(205)	0.11	64.9	(173)	0.85
YPP	2537	(182)	2612	(154)	0.088	75.4	(182)	
d-ROMs (U.CARR)								
Control	388	(95.7)	401	(85.0)	0.16	12.5	(37.7)	0.085
YPP	375	(108)	368	(89.5)	0.94	− 7.58	(32.6)	
BAP/d-ROMs								
Control	6.91	(1.46)	6.80	(1.34)	0.37	− 0.11	(0.54)	0.044*
YPP	7.18	(1.71)	7.44	(1.59)	0.064	0.27	(0.59)	
Ox-LDL (U/L)								
Control	93.7	(19.8)	79.4	(21.2)	< 0.001***	− 14.3	(10.0)	0.085
YPP	93.9	(20.1)	86.2	(21.7)	0.016*	− 7.79	(12.7)	
LPO (nmol/mL)								
Control	2.77	(0.26)	2.87	(0.37)	0.11	0.11	(0.28)	< 0.001***
YPP	2.74	(0.34)	2.49	(0.21)	< 0.001***	− 0.25	(0.23)	
8-OHdG (ng/mg crea)								
Control	6.66	(1.95)	7.29	(2.36)	0.20	0.64	(2.15)	0.086
YPP	6.29	(2.71)	5.61	(2.19)	0.25	− 0.69	(2.54)	
Isoprostane (ng/mg crea)								
Control	2.69	(1.38)	2.59	(0.90)	0.63	− 0.10	(0.92)	0.69
YPP	2.31	(1.02)	2.16	(0.74)	0.42	− 0.15	(0.81)	
HEL (pmol/mg crea)								
Control	92.6	(153)	75.0	(77.5)	0.50	− 17.6	(173)	0.12
YPP	55.8	(21.5)	42.5	(27.6)	0.15	− 13.3	(38.7)	

Data are presented as mean (SD). ^1^Within-group comparisons (Week 0 vs. Week 4) using paired-samples *t*-test or Wilcoxon signed-rank test. ^2^Group comparison of change using independent-samples *t*-test or Wilcoxon rank-sum test. *p < 0.05, **p < 0.01, ***p < 0.001. BAP, Biological antioxidant potential; d-ROMs, Diacron-reactive oxygen metabolites; LPO, Lipid peroxide; Ox-LDL, Oxidative low-density lipoprotein; 8-OHdG, 8-Hydroxy-2′-deoxyguanosine; HEL, Hexanoyl-lysine; YPP, Yellow pea pasta.

### Inflammation and glycation stress markers

Table 4 shows the biomarkers related to glycation stress and inflammation at Weeks 0 and 4 in the control and YPP groups. The pentosidine level increased significantly in the control group (*p* = 0.0035), whereas it did not significantly change in the YPP group (*p* = 0.68). No significant difference in the variation of this parameter was found between the two groups (*p* = 0.15). No significant change in the tumor necrosis factor-α (TNF-α) level was observed in the control group, whereas it significantly increased in the YPP group in Week 4 (*p* = 0.0073). No significant difference in the variation of this parameter was found between the two groups (*p* = 0.42). Neither group showed significant changes in the 3-deoxyglucosone (3-DG), HS-CRP, monocyte chemotactic protein 1 (MCP-1), or interferon-γ (IFN-γ) levels, and no significant differences in the variations of these parameters were found between the two groups.

**Table 4:** Inflammation and glycation stress markers.

	Week 0		Week 4		*p*^1^	Change		*p*^2^
Pentosidine (pmol/mL)								
Control	23.4	(5.02)	25.8	(5.39)	0.0035**	2.41	(3.23)	0.15
YPP	24.9	(8.00)	26.0	(8.51)	0.68	1.11	(2.06)	
3-DG (ng/mL)								
Control	175	(52.9)	166	(39.6)	0.26	− 8.35	(31.9)	0.070
YPP	176	(72.1)	189	(80.9)	0.17	13.2	(39.9)	
HS-CRP (mg/dL)								
Control	0.060	(0.074)	0.056	(0.077)	0.66	− 0.004	(0.088)	0.31
YPP	0.053	(0.056)	0.031	(0.033)	0.29	− 0.022	(0.036)	
MCP-1 (pg/mL)								
Control	157	(36.6)	166	(30.7)	0.051	9.05	(19.4)	0.080
YPP	197	(154)	195	(149)	0.86	− 1.68	(17.8)	
TNFα (pg/mL)								
Control	7.90	(3.79)	8.71	(4.26)	0.24	0.81	(2.48)	0.42
YPP	6.11	(1.91)	7.52	(2.89)	0.0073**	1.41	(2.04)	
IFN-γ (pg/mL)								
Control	5.00	(2.68)	6.45	(3.54)	0.30	1.45	(3.64)	0.38
YPP	5.55	(2.98)	6.12	(2.42)	0.30	0.57	(2.31)	

Data are presented as mean (SD). ^1^Within-group comparisons (0 Week vs. 4 Week) using paired-samples t-test or Wilcoxon signed-rank test. ^2^Group comparison of change using independent-samples t-test or Wilcoxon rank-sum test. *p < 0.05, **p < 0.01, ***p < 0.001. 3-DG, 3-Deoxyglucosone; HS-CRP, High-sensitivity C-reactive protein; MCP-1, Monocyte chemotactic protein 1; TNF-α, Tumor necrosis factorｰα; IFN-γ, Interferon-γ; YPP, Yellow pea pasta.

### Glycemic parameters

Table 5 shows the main parameters for 24-h blood glucose variations measured using continuous glucose monitoring (CGM) on days 2, days 8, days 15, days 22 and 28 in the control and YPP groups. On days 2, the maximum glucose concentration (Max) and mean amplitude of glycemic excursions (MAGE) was significantly lower in the YPP group than that in the control group (*p* = 0.045, 0.012); however, no significant differences were observed at any other time point. On days 2, days 8, days 15, days 22 and 28 no significant differences in the mean 24 h glucose concentration (Average), difference between the maximum and minimum glucose levels (ΔMax − Min), glucose standard deviation (Glucose SD), percentage coefficient of variation (%CV), or time in range (TIR) were observed between the two groups.

**Table 5:** Glycemic parameters.

	day2			*p*	day8				*p*		day15						*p*		day22						*p*		day28						*p*	
	n				n						n								n								n							
Average (mg/dL)																																		
Control	18	96.0	(18.1)	0.77	17	103	(16.5)		0.64		19		89.2		(12.7)		0.91		17		94.9		(13.0)		0.46		16		92.4		(15.0)		0.36	
YPP	19	94.5	(12.1)		19	100	(12.0)				19		89.7		(8.81)				18		92.2		(7.73)				16		88.2		(10.1)			
Max (mg/dL)																																		
Control	18	175	(28.7)	0.045*	17	164	(29.9)		0.35		19		134		(24.6)		0.12		17		152		(30.0)		0.68		16		143		(24.7)		0.29	
YPP	19	156	(28.5)		19	156	(19.5)				19		146		(22.6)				18		148		(25.8)				16		133		(23.6)			
ΔMax-Min (mg/dL)																																		
Control	18	119	(22.0)	0.053	17	88.5	(27.6)		0.29		19		73.0		(20.1)		0.078		17		84.7		(26.3)		0.65		16		77.7		(24.1)		0.22	
YPP	19	101	(31.2)		19	79.7	(20.6)				19		85.7		(23.0)				18		80.4		(28.4)				16		67.5		(22.2)			
Glucose SD (mg/dL)																																		
Control	18	25.1	(7.05)	0.30	17	21.2	(6.07)		0.069		19		17.1		(6.33)		0.77		17		20.1		(7.63)		0.29		16		17.9		(6.00)		0.24	
YPP	19	22.0	(7.52)		19	17.8	(4.39)				19		17.7		(5.04)				18		18.0		(7.14)				16		15.6		(4.83)			
MAGE (mg/dL)																																		
Control	18	67.0	(20.8)	0.012*	17	55.2	(18.3)		0.11		19		41.2		(17.2)		0.37		17		54.4		(24.6)		0.22		16		44.6		(16.2)		0.23	
YPP	19	49.3	(20.0)		19	46.5	(12.8)				19		46.0		(15.3)				18		46.7		(25.1)				16		37.9		(15.1)			
%CV (%)																																		
Control	18	26.6	(6.89)	0.17	17	20.7	(5.91)		0.12		19		19.0		(4.48)		0.60		17		20.9		(5.58)		0.35		16		19.5		(6.01)		0.42	
YPP	19	23.3	(7.28)		19	17.9	(4.33)				19		19.8		(5.70)				18		19.5		(7.56)				16		17.8		(5.81)			
TIR (%)																																		
Control	18	87.5	(13.5)	0.31	17	89.8	(15.4)		0.40		19		95.7		(8.88)		0.25		17		94.3		(10.4)		0.48		16		96.0		(10.9)		0.98	
YPP	19	26.6	(8.83)		19	94.0	(8.17)				19		96.8		(2.91)				18		96.5		(5.00)				16		96.2		(9.10)			

Data are presented as mean (SD). Group comparison at each day independent-samples *t*-test or Wilcoxon rank-sum test. *p < 0.05. SD, Standard deviation; MAGE, Mean amplitude of glycemic excursions; TIR, Time in range; YPP, Yellow pea pasta.

## Discussion

This exploratory study examined the effects of the YPP intake on the salt intake level, glycemic parameters, and blood oxidation markers in healthy men and women whose salt intake level exceeded the target salt equivalent level of the Japanese dietary intake standard.

The YPP intake improved several oxidative stress markers, based on the BAP/d-ROMs ratio and LPO level results. The power for the parameters for which no significant differences were observed, comparison of intergroup comparison of the variation in the d-ROMs and the 8-OHdG level, were 0.40, and 0.40, respectively. Considering the insufficient power, our results suggest that the YPP intake consistently improved oxidative stress in the participants. The YPP consumed by participants in this study contained 9.9–13.6 g of total dietary fiber per serving. Although fiber-induced changes in the gut microbiota of YPP may have contributed to the improvement in oxidative stress levels^18^, previous reports have shown that yellow pea fiber did not significantly alter the gut microbiota^19^. We also investigated the effect of YPP intake on the gut microbiota with similar result^20^. Several studies have demonstrated the antioxidant effect of pea hull polyphenols. The administration of the methanol extract of green pea hulls resulted in a decrease in the LPO (malondialdehyde) level in rat models of oxidative stress^21^. Similarly, the administration of the methanol extract of yellow pea hulls resulted in a decrease in the LPO (malondialdehyde) level in rat models of oxidative stress^22^. Furthermore, a study investigated the action mechanism of green pea hull polyphenols in dextran sulfate sodium-induced colitis mouse models and reported that they exert antioxidant and anti-inflammatory effects by activating the Keap1-Nrf2-ARE signal transduction pathway and regulating its downstream antioxidant enzymes^23^. In addition to these reports on the antioxidant effect of pea hull methanol extract, the intake of hulls themselves by non-alcoholic fatty liver mouse models improved the NAFLD level, inducing an antioxidant effect^24^. The main mechanism supporting the effect of the YPP intake in improving oxidative stress is likely the antioxidant effect of the polyphenols found in pea hulls. However, all these studies were conducted in animal models. The present study is the first to investigate the antioxidant effect of yellow peas in a clinical setting and demonstrated the effect of consuming pasta made exclusively from yellow peas, rather than their extract. The YPP consumed by participants in this study contained 72 mg of total polyphenols per serving. The main polyphenols found in yellow pea hulls are catechin, kaempferol, and quercetin^22^; however, the present study was unable to identify which fractions or molecules were the most effective. The identification and quantification of the polyphenols contained in YPP, as well as the examination of the detailed action mechanism of YPP, are expected to lead to further enhancement of the antioxidant effect of YPP in the future.

The anti-inflammatory effects reported in previous studies were demonstrated mainly using inflammation-induced animal models. Here, we measured the levels of inflammatory markers, TNF-α levels showed significant differences when comparing Week 0 to Week 4 in the YPP group. However, no consistent anti-inflammatory effect was clearly found since the changes identified were within the range of physiological variations^25^. The lack of marked variations in the levels of these markers is to be expected as this study targeted healthy participants. Conversely, the observation of a clear antioxidant effect suggests that the levels of inflammatory stress markers in human participants with advanced inflammation may be reduced through long-term YPP intervention. Further studies are needed to investigate this hypothesis.

We have previously reported that YPP enhances the sensitivity to saltiness^16^, and this study investigated whether the intake of YPP actually affects the salt intake in human participants. After four weeks of YPP intake, both the control and YPP intake groups showed a decrease in the salt intake level, according to the BDHQ results. To calculate the salt intake level in the BDHQ, the participants were asked to recall the past meals and answer the questionnaire. This likely prompted them to start better controlling their lifestyle and diet and have an increased awareness about reducing their salt intake, resulting in a decrease in the subjective salt intake level in both groups. Regarding the urine-based salt intake indices, however, only the control group participants showed a significant increase in the estimated 24-h urine Na^+^ excretion level, according to the spot urine collection test done after four weeks of YPP intake. Conversely, the salt intake level of the participants in the YPP group did not change during the study period. We believe that the use of both the questionnaire and actual measurements in this study enabled a more accurate evaluation of the salt intake level. No significant difference in the variation of this parameter was found between the two groups. However, considering the low power (0.32) obtained from our sample size, our results suggest that YPP may be effective in not increasing the salt intake. Certainly, a 24-h urine collection would lead to the most accurate evaluation of the salt intake level. However, this strategy was not used to avoid burdening the participants and to allow them to continue their normal lifestyle.

The sensory test in this study showed that the participants’ sensitivity to saltiness did not change even after four weeks of YPP intake. Individuals with a high salt intake level have a higher salt sensitivity threshold and are unable to feel satisfied unless they consume a larger amount of salt^26^. Therefore, to further reduce the salt intake level in real life, it is desirable to not only incorporate ingredients that enhance the salt sensitivity, such as YPP, into the diet, but also to use approaches that change both the salt sensitivity and palatability^27–29^.

We have previously reported that YPP, a low GI food^17^, suppresses postprandial insulin secretion^16^. Here, we used a CGM to investigate the degree of the effect of incorporating a meal composed of this low GI food on the increase in the blood glucose levels. To create a study setting that closely resembled real life, this study did not specify the timing or time of the YPP intake, and the participants were allowed to consume the food freely. Only a limited number of studies have evaluated a low GI food under conditions resembling a real-life scenario, and we believe that our study provides interesting new findings.

Our results showed significant differences in Max and MAGE on days 2 between the two groups; however, the YPP intake did not affect the other glycemic parameters. Moreover, YPP intake did not affect all glycemic parameters at other time points. This indicates that replacing a normal meal with a low GI food once a day does not necessarily result in a change in the blood glucose levels compared with those resulting from a normal diet without any intervention. The difference observed only in the Max and MAGE in the first 24 h may suggest the loss of the effect of YPP in lowering the blood glucose levels due to habituation; however, further investigation is required to validate this claim. Generally, trials using GI foods examine the degree of increase in the postprandial blood glucose levels compared to that resulting from the ingestion of glucose, white rice, and white bread, which tend to elevate the postprandial blood glucose levels^30^. Moreover, the intake of GI foods in combination with different cooking methods or side dishes has been investigated. The postprandial blood glucose levels can be affected by various food components that are consumed in our daily diet, such as fats, oils, proteins, and dietary fiber^31^. Therefore, the lack of changes in glycemic parameters observed in the present study may be because the participants were not in a state in which the blood glucose levels could easily and steadily rise, even in normal life conditions. In this study, the CGM sensor was replaced in the second week, and a few participants were unable to measure glucose via CGM in last 24 h due to poor sensor fit. Accuracy of measurement is a point of caution when measuring glucose using CGM in real-life.

Various metabolic diseases in patients with type II diabetes can be improved by incorporating low GI foods^32^. However, even if healthy individuals without metabolic abnormalities consume a meal containing low GI foods daily for the purpose of reducing the blood glucose levels, significant benefits in blood glucose control may not be observed. Moreover, despite our thorough investigation on the variations in the levels of glycation stress markers that are closely associated with blood glucose levels, none of these markers exhibited a decrease exclusively in the YPP group. This is likely due to the fact that the participants enrolled in this study had lower or same levels of glycation stress markers than that of those enrolled in previous studies of healthy individuals^33,34^. The pentosidine level significantly increased at the end of the study period in control group; however, this variation was thought to be within the physiological range for the reasons mentioned above. Although no effect of legume intake on the risk of metabolic diseases in healthy individuals has been demonstrated, legumes have been suggested to have a preventive effect on the associated risk factors^35^. In the future, the benefits of legume intake for the prevention of metabolic diseases may be demonstrated by using a nutritional epidemiology approach that compares consumers and non-consumers of legumes over several years and by conducting trials in which the criteria for subject selection are rigorously designed.

This study examined the antioxidant effect of YPP, as well as its effects on the salt intake and postprandial blood glucose levels, in a real-life setting. As a result, the effects on salt intake and blood glucose levels were limited. Furthermore, we observed a marked improvement in the levels of oxidative stress markers, which is likely attributed to the polyphenols and antioxidant components found in YPP, although it may also be associated with the slight improvement in the salt intake and postprandial blood glucose levels. Oxidative stress is thought to be involved in various diseases and pathologies, especially lifestyle-related diseases, and promotes atherosclerosis^36,37^. LPO indicates oxidative modification of lipids in cellular constituents and is known to be associated with increased cardiovascular risk^38^. BAP/d-ROMs has been explained to show the balance between the production of reactive oxygen species and antioxidant capacity; d-ROMs has been reported to be associated with chronic inflammation^39,40^. As inflammation is also associated with cardiovascular events^41^, improvements in these biomarkers may contribute to reducing the risk of cardiovascular disease. As the number of people with cardiovascular disease continues to increase worldwide^42^, YPP intake may help in the prevention and management of cardiovascular disease.

This study has several limitations. First, the Hawthorne effect could not be ruled out because this was an open trial in which non-intervention participants, rather than participants receiving a placebo, constituted the control group. We cannot rule out the possibility that the YPP intervention may have influenced participants' dietary habits and lifestyles, thereby altering their biomarkers. However, the study design did not allow for these changes to be accounted for. This is the major weakness of this study. A double-blind, parallel-group trial with a placebo should be conducted in the future to confirm the results obtained here. Second, this study had an exploratory trial design, and the sample size may not have been sufficient. Additionally, differences in some measurement items could not be fully visualized since this study targeted healthy individuals. We anticipate that future studies, with a larger sample size and clearly defined subject attributes, will provide more conclusive evidence for the efficacy of YPP.

In conclusion, our results show that replacing a normal meal with YPP once daily for four weeks significantly improved the oxidative stress marker levels. Together with our previous findings, this study provides evidence that pasta prepared from yellow peas is a promising functional staple food.

## Methods

### Study design

This open-label, parallel-group, randomized controlled trial was conducted at Ueno-Asagao clinic in Japan between October and December 2021. This was an exploratory study; therefore, no statistical settings could be established, and the sample size was set to 40 individuals. Participants were allocated to one of the following groups (n = 20): the group consuming one test meal per day (ZENB Noodles, ZENB Japan, Japan) and the group maintaining a normal diet. The independent TES Holdings Inc. allocation manager created a randomisation list with a block size of 4 using SAS version 9.4 (SAS Institute Inc., Cary, NC, USA). The participants were stratified using the following stratification factors: age at the preliminary test, salt intake level, according to the BDHQ (common medical interview sheet), and 24-h urine Na excretion level (mEq/day), which was assessed via spot urine collection. After randomly allocating the participants into two groups using stratified block randomization, we confirmed that no significant difference between the groups was found.

This study was conducted in accordance with the ethical principles of the Declaration of Helsinki, and the protocol was approved by the ethical review committees of Ueno-Asagao clinic and Mizkan Holdings Co., Ltd. (approval number: 21-E004). Participants were informed that participation was voluntary and that they would not suffer any disadvantage by not providing consent. All participants provided their written informed consent. This study was registered in the UMIN Clinical Trials Registry (15/10/2021 UMIN trial ID: UMIN000045752) and was conducted in accordance with the reporting statement of the Consolidated Standards of Reporting Trials (CONSORT) for randomized controlled research. We used the CONSORT checklist when writing our report^43^.

### Study participants

For subject recruitment, a telephone interview survey with volunteer Japanese registrants was conducted. A screening test was performed on 90 individuals who were eligible as study participants. Among those who met the selection criteria and did not meet the exclusion criteria who were deemed appropriate candidates by the principal investigator, 40 participants exhibiting high salt intake levels, as assessed using the BDHQ, and high estimated 24-h urine Na excretion (mEq/day), assessed via spot urine collection, were selected to participate in this study. The main selection criteria were as follows:

1. Healthy men and women aged ≥ 20 years and < 65 years at the time that they provided informed consent to participate in the study;
2. Those whose daily salt intake level exceeded the target salt equivalent level of the Japanese dietary intake standard (7.5 g/day for men and 6.5 g/day for women);
3. Those who ate three meals a day (breakfast, lunch, and dinner);
4. Those who had received sufficient explanation of the purpose and contents of this study, volunteered to participate after understanding the study, and were able to provide written consent to participate in the study;
5. Those who were able to visit the facility and participate in the tests on the designated test dates.

The main exclusion criteria were as follows: those who were unable to carry out the instructions given by the physicians during the intake period, those undergoing drug treatment (including medication for alcohol abuse), those undergoing drug treatment for the purpose of treating a disease in the past month, those who drink more than 60 g/day of pure alcohol equivalent on a daily basis and those with current or past serious illness (diabetes, dyslipidemia, cardiovascular disease, etc.).

### Data measurement

Participants underwent vital sign and physical measurements, blood tests, urinalysis, a survey on the salt intake level, which was done using the BDHQ, a taste survey on the saltiness (visual analogue scale [VAS] questionnaire), a brief dietary survey (for the purpose of confirming compliance with YPP intake), and a medical interview conducted by a doctor during clinic visits at Weeks 0 and 4. The glucose levels were measured daily from Week 0 until Week 4 using a CGM (FreeStyle Libre, Abbott Japan Co., Ltd., Tokyo, Japan).

#### Estimation of the salt intake level using the BDHQ

The participants were asked to answer the BDHQ, based on which salt intake level was calculated^44^.

#### Estimation of the salt intake level using a self-monitoring device

We used a monitoring device capable of estimating the salt intake level in the first-catch urine (KME-03, Kohno ME Laboratory, Japan). This device estimates the 24 h salt excretion level from an overnight urine sample using Eq. (1):

1 $$\text{Y}=1.95\text{X}+4.5\text{ g}/\text{day}$$

where Y is the estimated 24-h urine salt excretion level and X is the sodium content of the overnight urine sample^45^. The validity of this device for estimating the salt intake level has been previously demonstrated^46^. From the start of the study, the salt intake level from the meals of the previous day was estimated using a first-catch urine sample and recorded daily.

#### Vital sign and body composition measurements

At each clinic visit, the systolic blood pressure, diastolic blood pressure, and pulse were measured using an electronic blood pressure monitor (H55 Elemano, Terumo Corporation, Tokyo, Japan). The body composition data were measured using InBody770K (InBody Japan, Tokyo, Japan).

#### Blood sample

At each clinic visit, a blood sample (46.5 mL) was collected by a clinic nurse using a syringe and a blood collection tube. The participants were instructed to fast for at least 12 h before the scheduled blood sampling time and, in that period, they were only allowed to consume water. On the day of the test, participants were allowed to drink approximately 200 mL of water until the completion of the test and were instructed to refrain from drinking an excessive amount of water. The BAP (BAP Test, Wismerll Company Ltd., Tokyo, Japan) and d-ROMs (d-ROM Test, Wismerll Company Ltd.) were measured by the Olive Takamatsu Medical Clinic. The MCP-1 (Quantikine Human MCP-1 Immunoassay, R&D Systems, Inc., Minneapolis, MN, USA), TNF-α (QuantiGlo ELISA Human TNF-α Immunoassay QTA00C, R&D Systems, Inc.), IFN-γ (V-PLEX Proinflammatory Panel 1 Human Kit, Meso Scale Diagnostics, LLC., Rockville, MD, USA), aldosterone (Deteminer CL Aldosterone, Minaris Medical Co., Ltd., Tokyo, Japan), and pentosidine (Acquity UPLC H-CLASS, Waters Corporation, Milford, MA, USA) levels, and the activity of ACE1 (ACE color, Fujirebio Holdings, Inc., Tokyo, Japan), ACE2 (ACE2 (human) ELISA Kit, Adipogen Corporation, San Diego, CA, USA), and renin (Renin activity kit Yamasa, Yamasa Corporation, Chiba, Japan) in the plasma were measured by LSI Medience Co., Ltd. (Japan). The Ox-LDL (Oxidized LDL Elisa “DAIICHI,” Sekisui Medical Co., Ltd., Tokyo, Japan) and HS-CRP (Full-automatic immunochemistry analytical equipment BN II, Siemens Medical Solutions Inc., Tokyo, Japan) levels were measured by Nikken Seil Co., Ltd. (Japan). LPO were measured by Nikken Seil Co., Ltd. according to the report by Yagi et al.^47^. The 3-DG level was measured by AGELab Co., Ltd. (Japan) according to Yagi et al.^48^.

#### Urine sample

At each clinic visit, a 20 mL urine sample was collected. The Na^+^ was measured using an ion electrode method by Hoken Kagaku, Inc. (Japan). Creatinine levels was measured using an enzymatic method by Hoken Kagaku, Inc. The 24-h urine Na^+^ excretion, which was analyzed via spot urine collection, was calculated using Eqs. (2) and (3) ^49^:

2 $$\begin{aligned}&24\;{\hbox{h urine Na}}^{ + } {\hbox{excretion level}}\;\left({{\hbox{mEq}}/{\hbox{day}}} \right) = 21.98 \\ &\quad \times\left[ {\frac{{\frac{{{\hbox{Casual urine Na}}^{ + } \left({\frac{{{\hbox{mEq}}}}{{{\hbox{day}}}}} \right)}}{{{\hbox{Casual urine Cr }}\left( {\frac{{{\hbox{mg}}}}{{{\hbox{dL}}}}}\right)}}}}{10} \times {\hbox{Predicted }}24{\hbox{ h urine Cr excretion level }}\left( {{\hbox{mg}}/{\hbox{day}}} \right)}\right]^{0.392} \end{aligned} $$

3 $$ \begin{aligned} & {\text{Predicted 24 h urine Cr excretion level}}\;\left( {{\text{mg}}/{\text{day}}} \right) = {\text{Body weight }}\left( {{\text{kg}}} \right) \times 14.89 \\ & \quad + {\text{Height }}\left( {{\text{cm}}} \right) \times { }16.14 - {\text{Age}} \times { }20.4 - 2,244.45 \\ \end{aligned} $$

Estimated daily salt intake was calculated using Eq. (4):

4 $$\text{Estimated daily salt intake }\left(\text{g}/\text{day}\right)=\frac{24\text{ h urine }{\text{Na}}^{+}\text{excretion level }\left(\text{mEq}/\text{day}\right)}{17}$$

The 8-OHdG (New 8-OHdG Check, Nikken SEIL Co., Ltd.), Isoprostane (8-iso-PGF2α ELISA kit, Enzo Life Sciences, Inc., Farmingdale, NY, USA), and HEL (Hexanoyl-Lys adduct ELISA kit, Nikken SEIL Co., Ltd.) levels were measured by Nikken Seil Co., Ltd. and corrected based on the creatinine level.

#### Taste survey to assess the saltiness

A salt taste test was performed using a 100-mm VAS. Participants gargled with water first, ate soup with a known salt concentration, and answered the VAS questions. The answers of the participants were recorded based on a scale from 0, for those who sensed no saltiness, to 100, for those sensing a high level of saltiness.

#### Glucose measurement using a continuous glucose monitor

CGM (A FreeStyle Libre monitor, Abbott Japan Co., Ltd.) was inserted at the Week 0 clinic visit, and the sensor was replaced when the participants returned to the clinic in the second week. The sensor was removed at the clinic visit in Week 4. Data were collected at all clinic visits.

### Intervention

The participants in the YPP group were provided YPP and four flavors of pasta sauce (seven servings each; 28 servings in total) and consumed a combination of YPP and an arbitrarily chosen sauce as a substitute for a normal meal for any one of their three meals: breakfast, lunch, or dinner. This strategy was designed so that upon the completion of the 28-day intake period, the participants would have consumed all the YPP and sauce provided. The nutritional composition of the test meal is shown in Table 6. The total polyphenol content was measured using the Folin–Ciocalteu method^50^ and expressed as (+)-catechin equivalent values. If the flavor of the study food was undesirable for any participant, he/she was allowed to add seasonings, such as salt and pepper. Moreover, if the YPP and sauce alone did not induce a sense of fullness in any participant, they were permitted to consume foods other than these. The intake of additional foods was recorded on the questionnaire. Although we wanted to evaluate the potential health functions of YPP in this study, we were unable to prepare a high-quality placebo. For example, to evaluate the effect of salt reduction, it was necessary to prepare a placebo without umami components and salty-tasting peptides, and to evaluate the antioxidant effect of polyphenols, a placebo without polyphenols. Therefore, a control group consisting of participants who maintained their regular daily dietary habits was used for comparison.

**Table 6:** Nutritional composition of YPP per serving.

Per serving (80 g)	YPP
Energy (kcal)	261
Protein (g)	13.0–16.6
Fat (g)	0.9–2.5
Carbohydrate (g)	49.8–55.1
Total dietary fiber (g)	9.9–13.6
Total polyphenol (mg)	72

Total polyphenol is shown as (+)-catechin equivalents.

YPP, Yellow pea pasta.

### Outcomes

The outcomes of this study were the Salt intake level and related biochemical parameters, oxidative stress markers, Inflammation and glycation stress markers, constant blood glucose according to the CGM results, and the salt sensitivity, which was assessed via a taste test. The glucose levels measured using a CGM on days 2, days 8, days 15, days 22 and 28 of the study were evaluated to capture the blood glucose variations over a 24-h period. Some participants were unable to measure glucose by CGM due to poor sensor fit. Therefore, only data from participants who could be measured by CGM were evaluated. The following parameters related to blood glucose variations were determined for the entire 24 h period: Max, ΔMax − Min, glucose SD, MAGE, %CV, and TIR. The definitions and interpretations of each index have been described previously^51^. The MAGE was defined as the mean of the absolute values of the differences between the adjacent peak and that immediately below it, and all differences had at least an SD of 1. The %CV was calculated by dividing the SD by the mean blood glucose and was expressed as a percentage by multiplying it by 100. The TIR was defined as the time that the glucose level was kept within 70–180 mg/dL, the target glucose range for patients with diabetes^52^. Here, the cutoff value for healthy individuals was set to 54–140 mg/dL based on a previous report^53^.

### Statistical analysis

Data are expressed as the mean ± SD. Normality distribution of data was checked by visual inspections of histograms and by Shapiro-Wilks test. A paired t-test was used for intragroup comparisons of the participant data (Week 0 vs. Week 4). For data that were not normally distributed, a Wilcoxon signed-rank test was used instead. An unpaired t-test was used for the intergroup comparisons. For data that were not normally distributed, a Wilcoxon rank-sum test was used instead. We calculated post hoc power using Hedges’g^54^ for some data to examine the efficacy of YPP. Statistical analysis was performed using SAS version 9.4 (USA), IBM SPSS version 26 (IBM Corporation, Armonk, NY, USA), or R 4.3.0, and the significance level for all tests was set to 5% (*p* < 0.05). Since this was an exploratory trial study, no multiplicity adjustments were made.

## Supplementary Information

Supplementary Information.

## Data Availability

The datasets analyzed in the current study are not publicly available, as they cannot be made available due to lack of participant consent for data upload and for reasons of sensitivity but are available from the corresponding author upon reasonable request.

## Abbreviations

YPP: Yellow pea pasta
RAS: Renin-angiotensin system
NADPH: Nicotinamide adenine dinucleotide phosphate
AGE: Advanced glycation end-product
PKC: Protein kinase C
RAGE: Receptor for AGE
GI: Glycemic index
HS-CRP: High-sensitivity C-reactive protein
ACE: Angiotensin converting enzyme
BDHQ: Brief-type self-administered diet history questionnaire
BAP: Biological antioxidant potential
d-ROMs: Diacron-reactive oxygen metabolites
Ox-LDL: Oxidized low density lipoprotein
LPO: Lipid peroxide
8-OHdG: 8-Hydroxy-2′-deoxyguanosine
Iso: 15-Isoprostane F2t
HEL: Hexanoyl-lysine
TNF-α: Tumor necrosis factor-α
3-DG: 3-Deoxyglucosone
MCP-1: Monocyte chemotactic protein 1
IFN-γ: Interferon-γ
CGM: Continuous glucose monitoring
MAGE: Mean amplitude of glycemic excursions
Average: Mean 24 h glucose concentration
Max: Maximum glucose concentration
ΔMax-Min: Difference between the maximum and minimum glucose levels
SD: Standard deviation
%CV: Percentage coefficient of variation
TIR: Time in range
DSS: Dextran sulfate sodium
Keap1: Kelch-like ECH-associated protein 1
Nrf2: Nuclear factor-erythroid 2-related factor 2
ARE: Antioxidant response element
NAFLD: Non-alcoholic fatty liver disease
CONSORT: Consolidated standards of reporting trials
VAS: Visual Analogue Scale
