# Supplementary material for: Yellow pea-based pasta's impacts on the salt intake, glycemic parameters and oxidative stress in healthy individuals: a randomized clinical trial
Source: Sci Rep. 2024 Oct 7;14:23333. doi: 10.1038/s41598-024-72290-6 (PMC11458757; doi:10.1038/s41598-024-72290-6)
Supplement: Supplementary file 1 — Supplementary Information. [file 41598_2024_72290_MOESM1_ESM.docx]

**Yellow pea-based pasta's impacts on the salt intake, glycemic parameters and oxidative stress in healthy individuals: A randomized clinical trial**

Mamoru Ito^1,5*^, Joto Yoshimoto^1,5^, Sho Ishii^1^, Tetsuya Maeda^2^, Yu Wada^2^, Yoshikazu Yonei^3^, Mikiya Kishi^1^, and Takahiro Ono^4^

1 Central Research Institute, Mizkan Holdings Co., Ltd., Handa-Shi, Aichi, Japan

2 New Business Development, Mizkan Holdings Co., Ltd., Chuo-ku, Tokyo, Japan

3 Anti-Aging Medical Research Center, Faculty of Life and Medical Sciences, Doshisha University, Kyotanabe-Shi, Kyoto, Japan

4 Ueno-Asagao clinic, Taito-ku, Tokyo, Japan

5 These authors contributed equally to this work: Mamoru Ito and Joto Yoshimoto.

*Corresponding author　(email: mamoru_ito@mizkan.co.jp)

|  |  | Week 0 | |  | Week 4 | | *p*^1^ |  | Change | | *p*^2^ |
| --- | --- | --- | --- | --- | --- | --- | --- | --- | --- | --- | --- |
| Body Weight (kg) | Control | 64.0 | (11.9) |  | 63.7 | 11.5 | 0.22 |  | -0.28 | (1.01) | 0.64 |
|  | YPP | 62.2 | (12.5) |  | 62.1 | (12.2) | 0.55 |  | -0.14 | (0.97) |  |
| Muscle mass (kg) | Control | 43.8 | (10.4) |  | 43.8 | (9.82) | 0.68 |  | -0.075 | (0.80) | 0.41 |
|  | YPP | 43.5 | (10.1) |  | 43.7 | (9.80) | 0.44 |  | 0.126 | (0.70) |  |
| Lean mass (kg) | Control | 46.4 | (11.0) |  | 46.3 | (10.4) | 0.62 |  | -0.095 | (0.84) | 0.41 |
|  | YPP | 46.1 | (10.7) |  | 46.3 | (10.3) | 0.51 |  | 0.12 | (0.79) |  |
| Body fat mass (kg) | Control | 17.6 | (5.58) |  | 17.4 | (5.62) | 0.28 |  | -0.19 | (0.76) | 0.75 |
|  | YPP | 16.1 | (6.74) |  | 15.9 | (6.74) | 0.046^*^ |  | -0.26 | (0.52) |  |
| Percent body fat (%) | Control | 27.7 | (8.18) |  | 27.4 | (7.98) | 0.26 |  | -0.29 | (1.09) | 0.68 |
|  | YPP | 25.7 | (8.54) |  | 25.3 | (8.41) | 0.033^*^ |  | -0.41 | (0.77) |  |
| BMI (kg/m^2^) | Control | 23.5 | (3.07) |  | 23.4 | (3.02) | 0.29 |  | -0.090 | (0.37) | 0.85 |
|  | YPP | 22.7 | (3.72) |  | 22.7 | (3.63) | 0.40 |  | -0.068 | (0.35) |  |
| Visceral fat area (cm^2^) | Control | 80.2 | (37.8) |  | 77.5 | (39.0) | 0.27 |  | -2.70 | (10.6) | 0.23 |
|  | YPP | 87.9 | (49.0) |  | 81.0 | (49.1) | 0.035^*^ |  | -6.87 | (10.6) |  |
| SBP (mmHg) | Control | 75.6 | (9.60) |  | 69.1 | (11.3) | <0.001^***^ |  | -6.45 | (7.17) | 0.26 |
|  | YPP | 71.5 | (11.7) |  | 68.2 | (10.7) | 0.16 |  | -3.32 | (9.78) |  |
| DBP (mmHg) | Control | 120 | (11.1) |  | 116 | (13.4) | 0.094 |  | -4.30 | (10.9) | 0.39 |
|  | YPP | 118 | (13.5) |  | 111 | (12.1) | 0.0027^**^ |  | -7.11 | (8.91) |  |

**Supplementary Table S1.** Vital signs and body composition

Data shows means (SD).　
^1^ Within-group comparisons (Week 0 vs. Week 4) based on paired-samples t-test. ^2^ Group comparison of change based on independent-samples t-test. ^*^ *p* < 0.05, ^**^ *p* < 0.01, ^***^ *p* < 0.001. BMI, Body mass index; SBP, Systolic blood pressure; DBP, Diastolic blood pressure; YPP, Yellow pea pasta.

|  |  | n |  |  | *p* |
| --- | --- | --- | --- | --- | --- |
| Average (g/d) | Control | 20 | 9.77 | (2.31) | 0.26 |
|  | YPP | 19 | 8.99 | (1.89) |  |
| Male | Control | 11 | 10.7 | (2.31) | 0.061 |
|  | YPP | 10 | 8.61 | (2.11) |  |
| Famale | Control | 9 | 8.60 | (1.79) | 0.47 |
|  | YPP | 9 | 9.21 | (1.73) |  |
| Total (g) | Control | 20 | 273 | (65.0) | 0.26 |
|  | YPP | 19 | 251 | (52.3) |  |
| Male | Control | 11 | 300 | (64.7) | 0.054 |
|  | YPP | 10 | 245 | (57.6) |  |
| Famale | Control | 9 | 240 | (50.7) | 0.45 |
|  | YPP | 9 | 258 | (48.3) |  |

**Supplementary Table S2.** The average and total estimated salt intake using self-monitoring devices during the study period

Data shows Means (SD). *p* Group comparison based on independent-samples t-test. YPP, Yellow pea pasta
